# Skin Anti-Aging Potential of *Ipomoea pes-caprae* Ethanolic Extracts on Promoting Cell Proliferation and Collagen Production in Human Fibroblasts (CCD-986sk Cells)

**DOI:** 10.3390/ph15080969

**Published:** 2022-08-06

**Authors:** Tasanee Panichakul, Saranyoo Ponnikorn, Wipa Tupchiangmai, Woraphot Haritakun, Kitima Srisanga

**Affiliations:** 1 Department of Cosmetic Science, Faculty of Science and Technology, Suan Dusit University, 228-228/1-3 Sirindhorn Rd. Bangphlat, Bangkok 10700, Thailand; 2 Chulabhorn International College of Medicine, Thammasat University (Rangsit Center), 99 Moo 18, Paholyothin Rd. Klongnung, Klongluang, Rangsit 12120, Thailand or; 3 Department of Chemical Technology, Faculty of Science and Technology, Suan Dusit University, 228-228/1-3 Sirindhorn Rd. Bangphlat, Bangkok 10700, Thailand; 4 Department of Biochemistry, Faculty of Science, Mahidol University, 272 Rama VI Rd., Ratchathewi District, Bangkok 10400, Thailand

**Keywords:** *Ipomoea pes-caprae*, skin aging, antioxidant, collagenase, collagen, cell proliferation, anti-aging

## Abstract

Collagen loss in the skin dermis is a major cause of age-related changes to the skin. Natural phytochemical substances are desirable for the prevention of skin aging and the formation of wrinkles. *Ipomoea pes-caprae* (IPC) has been utilized for nutritional and therapeutic purposes, and its extract contains collagenase inhibitory activity while causing no cytotoxicity. The purpose of this study was to examine the impact of IPC extracts on cell proliferation and collagen production in human fibroblasts (CCD-986sk cells). IPC leaves were macerated in 70% and 95% ethanol and the chemical composition of the resulting extracts (IPC70 and IPC95) were determined using high performance liquid chromatography (HPLC). The bioactivity of IPC extracts was examined in CCD-986sk cells, including antioxidant capacity, inhibition of collagenase, effects on cell proliferation and collagen production, as well as wound healing using an in vitro scratch test. Changes in expression of collagen type I (COL1A1), tumor growth factor beta 1 (TGFB1), and beta-fibroblast growth factor (FGF2) genes were also evaluated. The antioxidant and collagenase inhibitory properties of IPC extracts were associated with 3,5-di-caffeoylquinic acid, chlorogenic acid, and ferulic acid. IPC extracts at noncytotoxic concentrations significantly increased cell proliferation, collagen production, and wound healing. These effects appear linked to the upregulation of COL1A1, TGFB1, and FGF2 genes. The bioactivity of the IPC70 extract was greater than that for IPC95. This is useful in cosmeceutical applications for human skin aging. Our findings indicate that IPC extracts have the potential for use in skin anti-aging cosmeceutical preparations.

## 1. Introduction

Skin aging is a complex biochemical process that is influenced by both intrinsic and extrinsic factors, such as age, hormone levels, and exposure to UV light [1]. The process of skin aging occurs in the epidermal and dermal layers and is primarily caused by the deterioration of the extracellular matrix (ECM) [2]. The enzymes involved in ECM degradation are matrix metalloproteinases (MMPs), including collagenase, elastase, and gelatinase [3]. ECM degradation by MMPs causes skin to lose its tensile strength, promoting the appearance of aging. Dermal fibroblasts are the most prominent cell type in the dermis and play a crucial role in the progression of skin aging through their ability to secrete large amounts of extracellular matrix (ECM) components, such as collagen, hyaluronic acid (HA), and elastic fibers [4]. Type I collagen is the major structural component of the extracellular matrix (ECM), which functions in the maintenance of the structure of the skin dermis [5]. Transforming growth factor-β1 (TGF- β1) and fibroblasts growth factor 2 (FGF2) promote type I collagen synthesis. The balance between degradation and synthesis determines both the quantity as well as the quality of extracellular collagen [6]. In addition to the ECM structure, cell proliferation and migration impact the process of skin aging. Biochemical communication through paracrine signaling or gap junctions contributes to cell migration and proliferation [7]. Cell proliferation and migration processes play important roles in tissue formation and repair, maintaining healthy skin [8].

There is increasing interest in the identification of natural phytochemicals capable of reducing skin aging or wrinkling. Plant extracts rich in phytochemicals, such as flavonoids, phenolic acids, saponins, and alkaloids are being investigated in the development of anti-skin-aging products due to their potential to promote cell proliferation and collagen synthesis [6]. Preparations of *Ipomoea pes-caprae* (IPC) are used in Thai folk medicine as an anti-pruritogenic agent to treat jellyfish and Portuguese man-of-war sting-induced dermatitis [9]. In addition, IPC preparations are used for the treatment of several other conditions, including cramps, diuretic disorder, gonorrhea, inflammation, and pain [10,11,12]. Several studies have reported biological activities of the extracts, fractions, and isolated compounds from IPC that include antioxidant, anti-inflammatory, antinociceptive, antimicrobial, collagenase inhibitory, antispasmodic, anticancer, antitumor and antiproliferative, and multidrug-resistance efflux inhibitory activities [13]. The biological activities of IPC extracts are generally related to the presence of phenols, flavonoids, tannins, alkaloids, sterols, terpenoids, norisoprenoids, and glycosides [13,14,15,16,17]. Recently, studies have demonstrated IPC leaf extract as a source of phenolic compounds, highlighting neochlorogenic, chlorogenic, and isochlorogenic acids that had anti-inflammatory, wound healing, and antiophidic properties [14]. Oleamide, amyrin, phytol, and friedelin from ethanol and hexane extracts of IPC had anti-inflammatory and detoxification activities [18]. Interestingly, IPC extracts contain isochlorogenic acids a, b, and c, as well as quinic acid esters, which have been shown to inhibit collagenase with little cytotoxicity, suggesting that IPC preparations could be used to prevent skin aging [19].

In this study, IPC leaf extracts were monitored for antioxidant and collagenase inhibitory activities as well as for their potential to prevent extracellular matrix degradation. In addition, the potential of IPC extracts to promote human fibroblast proliferation and migration in vitro and enhanced wound healing was evaluated. Elevated expressions of collagen type I (COL1A1), tumor growth factor-beta 1 (TGFB1), and beta-fibroblast growth factor (FGF2) genes were associated with increased collagen production. The results presented indicate that IPC extract has the potential for use in cosmeceutical applications to reduce the effects of skin aging.

## 2. Results

### 2.1. Chemical Profiles, Total Phenolic, Flavonoid, Tannin Contents of Ipomoea pes-caprae Extracts

The total phenolic, flavonoid, and tannin contents of IPC extracts prepared using 70% or 95% ethanol (IPC70 and IPC95) were determined. The total phenolic contents of IPC70 and IPC95 extracts were 75.315 ± 9.074 and 67.095 ± 6.202 mg gallic acid equivalent (GAE)/g extract, respectively. The results indicated no significant differences in the total phenolic content between IPC70 and IPC95 preparations. The total flavonoid content from the IPC95 extract (26.815 ± 1.133 mg quercetin equivalent (QE)/g extract) was significantly higher than that in the IPC70 (17.641 ± 0.966 mg GE/g extract) (*p* < 0.05). In contrast, the total tannin contents of the IPC70 and IPC95 were 18.39 ± 0.77 and 6.57 ± 0.68 mg tannic acid equivalent (TAE)/g extract, respectively (Table 1). These results indicate that the phenolic compounds were the major content obtained with the ethanol extraction, and ethanol different concentrations had a significant impact on flavonoid and tannin contents.

**Figure 1:**
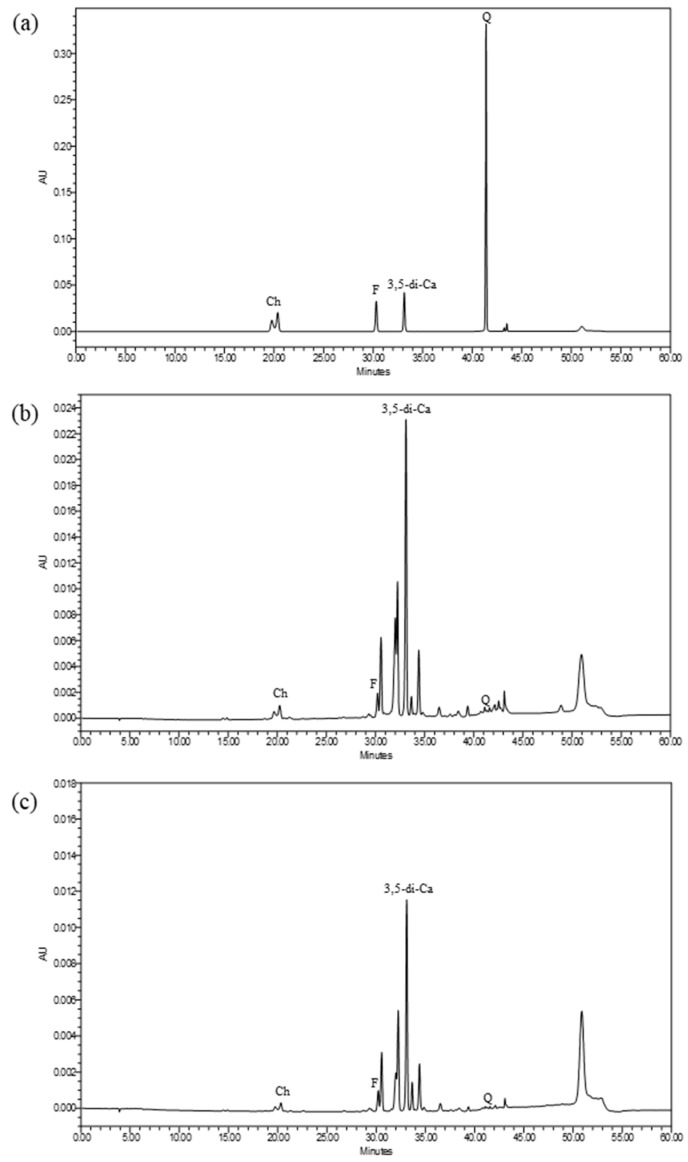
Chromatograms from HPLC analysis of IPC70 and IPC95 extracts. Chlorogenic acid (Ch), ferulic acid (F), 3,5-di-caffeoylquinic acid (3,5-di-Ca), and quercetin (Q) were detected at 360 nm. (**a**) standards, (**b**) IPC70 extract, and (**c**) IPC95 extract.

Several chemical constituents from IPC70 and IPC95 extracts were identified using high performance liquid chromatography (HPLC) analysis (Figure 1). Chemical compounds found in IPC70 and IPC95 extracts included chlorogenic acid (RT = 20.343 min), ferulic acid (RT = 30.219 min), 3,5-di-caffeoylquinic acid (RT = 33.098 min), and quercetin (RT = 41.347 min). The major compound found in both IPC70 and IPC95 extracts was 3,5-di-caffeoylquinic acid (Figure 1b,c). The concentrations of chlorogenic acid, ferulic acid, 3,5-di-caffeoylquinic acid, and quercetin in IPC70 extract were 7.53 ± 0.286, 6.646 ± 0.303, 54.19 ± 3.118, and 1.072 ± 0.078 mg/g of extract, respectively (Figure 1b). For IPC95 extracts, the concentrations of chlorogenic acid, ferulic acid, 3,5-di-caffeoylquinic acid, and quercetin were 4.59 ± 0.118, 4.398 ± 0.054, 29.43 ± 0.23, and 0.982 ± 0.006 mg/g of extract, respectively (Figure 1c). This analysis indicated that the contents of 3,5-di-caffeoylquinic acid, chlorogenic acid, and ferulic acid in IPC70 extracts were significantly higher than in IPC95 extracts (*p* < 0.05).

### 2.2. Antioxidant and Collagenase Inhibitory Activities of Ipomoea pes-caprae Extracts

The antioxidant activities of IPC70 and IPC95 extracts were examined using DPPH, ABTS, and FRAP analyses. The results are presented as the concentrations that inhibited 50% of the antioxidant activity (IC_50_) for DPPH and ABTS analyses, while the results for FRAP analysis are reported as µM Trolox equivalent (TE)/g extract. IPC70 was able to scavenge DPPH and ABTS radicals with IC_50_ values of 0.342 ± 0.021 and 0.259 ± 0.070 mg/mL, respectively. The IPC70 extract exhibited elevated antioxidant activity compared to IPC95 (0.403 ± 0.036 and 0.601 ± 0.057 for DPPH and ABTS, respectively). Values for the FRAP analysis also indicated that IPC70 had higher antioxidant activity compared to IPC95 (170,427.67 ± 1325.24 and 53,530.40 ± 4587.89 µM TE/g extract, respectively). The inhibitory effects of IPC70 and IPC95 extracts on collagenase also indicated a higher potency for IPC70 compared with IPC95, with IC_50_ of 5.541 ± 0.044 and 12.179 ± 0.413 mg/mL, respectively (Table 2). These results suggest that IPC extracts possessed antioxidant and collagenase inhibitory activities. In addition, IPC extracted with 70% ethanol exhibited higher antioxidant and collagenase inhibitory activities than the 95% ethanolic extract.

### 2.3. Cytotoxicity of Ipomoea pes-caprae Extracts on Human Fibroblasts (CCD-986sk Cells)

**Figure 2:**
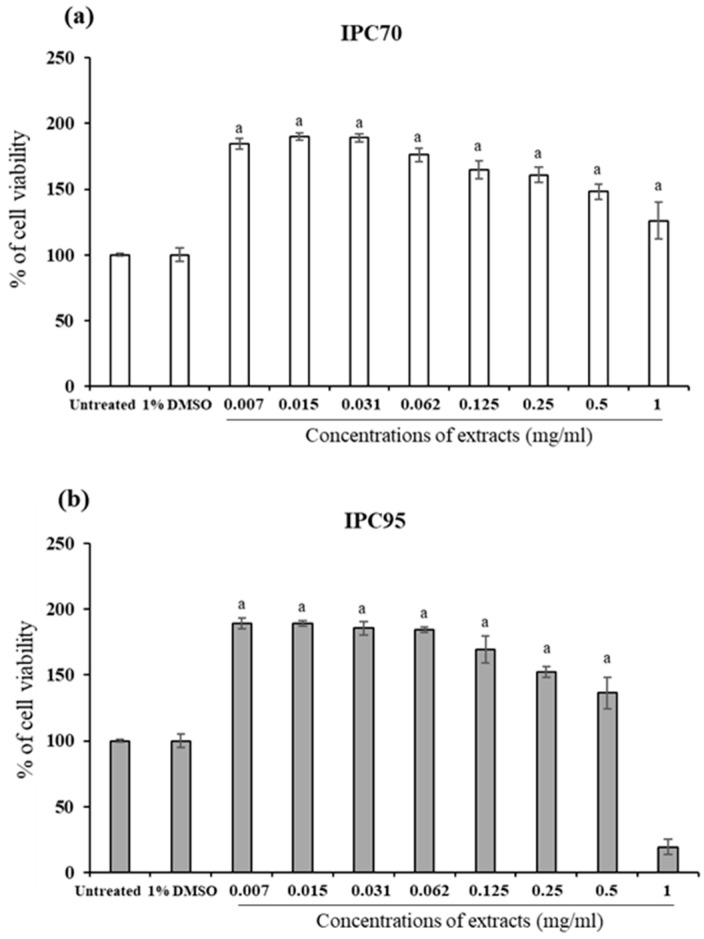
Cell viability of human fibroblasts (CCD-986sk cells) treated with *I. pes-caprae* extracts. (**a**) IPC70 and (**b**) IPC95 extracts. The percent cell viability is reported as mean ± SD from three independent experiments. The untreated control was set at 100%; (^a^) indicates a significant difference at *p* < 0.05.

Cytotoxicity of IPC extracts was examined using the PrestoBlue^®^ cell viability reagent to identify non-cytotoxic concentrations of the IPC70 and IPC95 extracts (Figure 2). Survival of more than 99% with CCD-986sk cells was used to determine the safe concentration of IPC70 and IPC95 extracts. After treatment for 72 h, the concentrations of IPC70 (0.007–1 mg/mL) and IPC95 (0.007–0.5 mg/mL) were found to have no cytotoxic effect on CCD-986sk cells (Figure 2a,b). These concentrations of IPC70 and IPC95 extracts were considered safe. The analysis of effects of IPC extracts on cell proliferation, wound healing, induction of gene expression, and collagen production used concentrations in the range established as non-cytotoxic.

### 2.4. Ipomoea pes-caprae Extracts Induced Cell Proliferation

**Figure 3:**
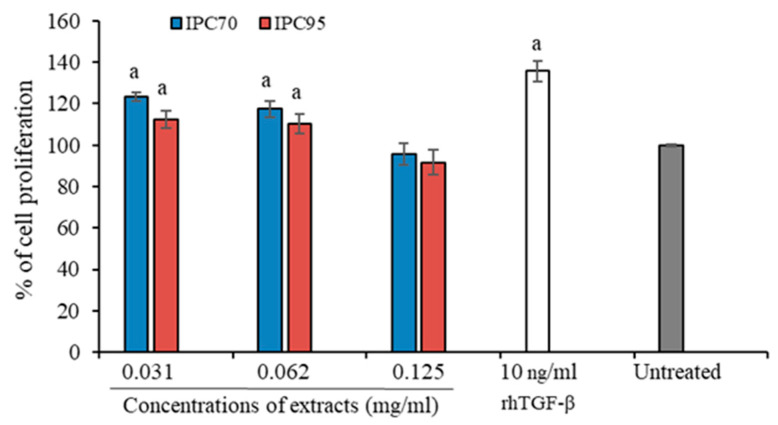
Cell proliferation of CCD-986sk cells treated with IPC70 and IPC95 extracts and rhTGF-β for 72 h. Values for cell proliferation are presented as the mean ± SD from three independent experiments. The untreated control was set at 100%; (^a^) indicates a significant difference at *p* < 0.05.

The effect of IPC extracts on the proliferation of CCD-986sk cells was examined using the PrestoBlue^®^ cell viability reagent. Cell viability above 100% of untreated CCD-986sk cells was considered cell proliferation. As shown in Figure 3, the proliferation of CCD-986sk cells was significantly increased after treatment with 10 ng/mL of rhTGF-β (135.66 ± 6.33%) compared to untreated cells. CCD-986sk cells treated with 0.031 and 0.062 mg/mL of IPC70 exhibited 123.32 ± 3.68 and 117 ± 4.64% cell viability, respectively, indicating induced cell proliferation. Cells also displayed proliferation after treatment with 0.031 and 0.062 mg/mL of IPC95 with 112.35 ± 4.65 and 110.32 ± 5.68% cell viability, respectively. The proliferation of CCD-986sk cells was not increased significantly at a concentration of 0.125 mg/mL of IPC70 and IPC95 extracts. These results indicate that 0.031 and 0.062 mg/mL of both IPC70 and IPC95 were able to induce cell proliferation compared with the untreated control.

### 2.5. Ipomoea pes-caprae Extracts Induced In Vitro Wound Healing

**Figure 4:**
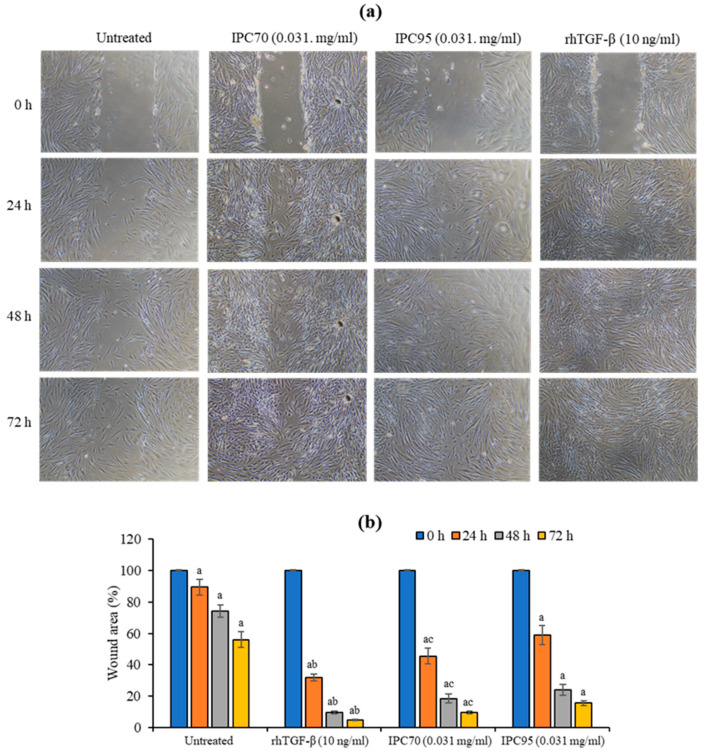
In vitro wound healing of CCD-986sk cells treated with IPC70 and IPC95 extracts. (**a**) Scratch wounds were observed using an inverted light microscopic (magnification ×100). (**b**) The percentage of cell-free or wound area was measured using the Image J program. The data are means ± SD from three independent experiments. The superscript letters (^a,b,c^) indicate a significant difference at *p* < 0.05; (^a^) treated cells at 24, 48, and 72 h were compared with treated cells at 0 h; (^b^) rhTGF-β -treated cells were compared with IPC-treated cells; (^c^) IPC70-treated cells were compared with IPC95-treated cells.

The in vitro scratch wound assay allows the assessment of cell movement or migration. Treatment with IPC extracts significantly accelerated wound closure compared with cells at 0 h Figure 4a. The wound area was reduced after 72 h of treatment with 10 ng/mL of rhTGF-β by 4.83 ± 0.57%. Treatment with 0.031 mg/mL of IPC70 and IPC95 extracts reduced the wound area by 9.76 ± 0.91 and 15.68 ± 1.52%, respectively, compared to cells at 0 h. Treatment with rhTGF-β, IPC70, and IPC95 significantly promoted wound recovery compared with untreated cells. The effect on wound recovery was the greatest for rhTGF-β. IPC70 showed a significant increase in wound healing compared to IPC95 (Figure 4b), indicating that IPC was capable of promoting cell movement or migration to fill the wound or gap area.

### 2.6. Effect of Ipomoea pes-caprae Extracts on Production of Type I Collagen and Expression of COL1A1, TGFB1, and FGF2 Genes

**Figure 5:**
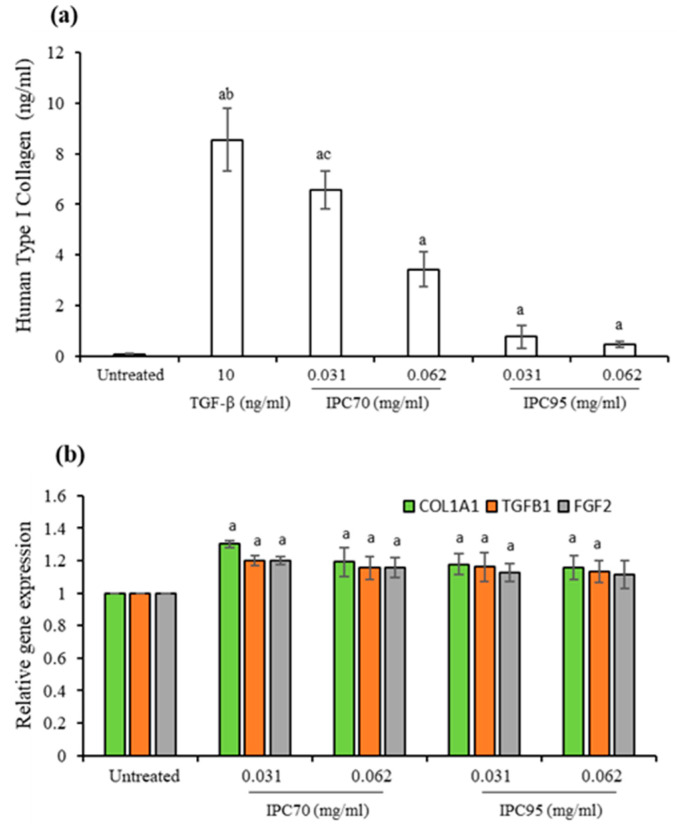
Effect of *I. pes-caprae* extracts on production of type I collagen and expression of COL1A1, TGFB1, and FGF2 genes. (**a**) Type I collagen production by CCD-986sk cells. (**b**) Expression of COL1A1, TGFB1, and FGF2 genes in CCD-986sk cells. Values are normalized to GAPDH and expressed relative to the values of untreated cells. The data are means ± SD from three independent experiments. The superscript letters (^a,b,c^) indicate significant difference at *p* < 0.05. (^a^) treated cells were compared with untreated cells; (^b^) rhTGF-β -treated cells were compared with IPC-treated cells; (^c^) IPC70-treated cells were compared with IPC95-treated cells.

The effects of IPC extract on type I collagen production in CCD-986sk cells were determined using a sandwich enzyme immunoassay [20]. As shown in Figure 5a, the production of type I collagen was increased significantly compared with untreated cells, following treatment with rhTGF-β and IPC extracts (*p* < 0.05). Type I collagen levels after treatment with 10 ng/mL of rhTGF-β, and 0.031 and 0.062 mg/mL of IPC70 were 8.55 ± 1.25, 6.55 ± 0.75 and 3.43 ± 0.68 ng/mL, respectively. The highest level of type I collagen was produced with the rhTGF-β treatment, while IPC95 exposed cells produced the lowest levels of type I collagen. IPC70 enhanced the production of type I collagen to a greater level than IPC95.

The expression of COL1A1, TGFB1, and FGF2 genes was monitored in CCD-986sk cells treated with IPC extracts. Compared to untreated cells, IPC70 (at concentrations of 0.031 and 0.062 mg/mL) and IPC95 extracts (at a concentration of 0.031 mg/mL) significantly upregulated the mRNA levels of these genes (*p* < 0.05), as shown in Figure 5b. The upregulation of the COL1A1 gene, encoding the pro-alpha1 chain of type I collagen [21], was consistent with elevated levels of type I collagen following exposure to IPC extracts. Expression of TGFB1 is involved in wound healing by modulating extracellular matrix formation and FGF2 encoding growth factors that stimulate cellular proliferation [22,23]. Enhanced expression of TGFB1 and FGF2 in cells treated with IPC extracts appears to be correlated with increased wound healing and cell proliferation.

Taken together, our findings indicate that IPC extracts are able to reduce free radicals, inhibit collagenase, and promote cell proliferation and migration to repair wound areas. In addition, treatment with IPC extracts induces the expression of COL1A1, TGFB1, and FGF2 genes, promoting collagen production in human fibroblasts. IPC70 exhibited more potent bioactivity compared to the IPC95 extract.

## 3. Discussion

Our findings provide the first report on the ability of *I. pes-caprae* (IPC) extracts to promote cell proliferation, wound healing, and collagen production, as well as induce expressions of COL1A1, TGFB1, and FGF2 genes in human fibroblast CCD-986sk cells. In addition, the IPC extracts also significantly scavenged free radicals and inhibited collagenase activity.

Reactive oxygen species (ROS) are thought to play a key role in dermal extracellular matrix alterations from both intrinsic aging and photoaging. The MAPK pathway is activated by ROS, which leads to an increase in MMP synthesis, promoting collagen degradation. Detoxification of ROS by antioxidative enzymes, such as superoxide dismutase, catalase, glutathione peroxidase, and coenzyme Q10, can protect against aging [24,25]. ROS-mediated effects on aging can also be reduced by treatment with antioxidants, such as vitamin C and vitamin E. In addition, plants are a natural source of antioxidants, and several studies have shown powerful antioxidant activity in the extract of *I. pes-caprae* leaves [13,26,27,28,29,30]. Our results demonstrate that the major phytochemicals present in the IPC leaf extracts were phenolic compounds, flavonoids, and tannins. The major phenolic component in the IPC extract was 3,5-di-caffeoylquinic acid. IPC extracts exhibited antioxidant activity based on DPPH- and ABTS-scavenging and ferric-reducing abilities. Total phenolic and flavonoid levels are strongly correlated with antioxidant potential, indicating that *I. pes-caprae* is a natural source of antioxidants. Tannins were also present in IPC extract, and these compounds possess hyaluronidase inhibitory activity [31]. Interestingly, IPC extracts also exhibit collagenase inhibitory activity, which can play a vital role in suppressing wrinkle formation. Previously, phenolic compounds, isochlorogenic acid a, isochlorogenic acid b, and isochlorogenic acid c, from *I. pes-caprae* were reported to inhibit collagenase activity [19]. In our study, 3,5-di-caffeoylquinic acid was found to be a major component of IPC ethanolic extracts. This compound was previously reported as isochlorogenic acid a and exhibited collagenase inhibitory activity [19,26]. Chlorogenic acid and ferulic acid were also found in IPC extracts, and these components may contribute to the collagenase inhibitory activity. Chlorogenic acid found in extracts from Hypericum androsaemum L red berries exhibits collagenase inhibitory activity [32]. Ferulic acid has also been shown to have antioxidant and collagenase inhibitory properties [33].

Wound healing, which often accompanies various skin conditions, is a complex multifactorial mechanism [34]. In the early stages of wound healing, fibroblasts play an extremely important role. Proliferation and migration to the wound area induce the synthesis of a new extracellular matrix [35]. This effect is associated, among others, with the increase in type 1 collagen expression [36]. Collagen is the major foundation of the extracellular matrix (ECM) in the dermis that imparts tensile strength to the human skin [37,38]. The structural characteristics of collagen in the dermis are closely related to skin aging, and the degradation of collagen in the dermis promotes a wrinkled appearance [39]. In addition, type I collagen can act as a ligand for receptor-mediated signaling to regulate many aspects of cellular processes downstream, including migration, cell survival, and growth [40]. Therefore, the assessment of the migration and proliferative capacity of fibroblasts is crucial in the search for compounds of natural origin that could support wound-healing processes and enhance type 1 collagen expression. Our results indicate that IPC extracts at noncytotoxic concentrations (0.031 mg/mL) significantly enhanced the proliferation of fibroblasts. The scratch wound test, a measure of cell migration and improved wound healing, demonstrated that treatment with IPC extracts for 24–72 h was capable of enhancing the proliferation and migration of fibroblasts filling the wound area.

The production of type I procollagen in intrinsically aged human skin is reduced, likely due to decreased TGF-b/Smad signaling [41]. TGF-β is known to stimulate fibroblasts in the dermis to synthesize collagen, the main constituent of dermal ECM [42]. TGF-β-induced fibrotic stress is also associated with increased expression of type 1 collagen [43]. The IPC extracts were able to induce the expression of TGFB1, COL1A1, and FGF2 genes, which are essential for fibroblast proliferation, collagen production, and wound healing. A possible mechanism for the anti-aging potential of IPC is through the production of human collagen type I mediated by the induction of the TGF-beta-signaling pathway. However, the underlying mechanism of IPC in promoting cell proliferation and migration in filling the wound area and inducing collagen production will require further investigation. In addition, primary human dermal fibroblasts from elderly donors are one of the notable alternatives in the study of IPC extracts.

Our results indicate that the *I. pes-caprae* extracts have antioxidant and inhibited collagenase inhibitory activities, which play a critical role in preventing collagen breakdown. This extract also dramatically induced human fibroblast proliferation and enhanced cellular collagen production. Therefore, *I. pes-caprae* leaf extracts appear to be effective in limiting the effects of skin aging and may promote skin repair mechanisms.

## 4. Materials and Methods

### 4.1. Chemicals

Folin–Ciocalteu’s Phenol reagent, 2,2-diphenyl-1-picrylhydrazyl (DPPH), 2,2′-Azinobis-(3-ethylbenzo-thiazoline-6-sulfonic acid) (ABTS+), Ferric chloride hexahydrate, 2,4,6-Tris (2-pyridyl)-s-triazine (TPTZ), sodium bicarbonate, tannic acid, Trolox, gallic acid, quercetin, chlorogenic acid, ferulic acid, and 3,5-di-caffeoylquinic acid were purchased from Sigma-Aldrich, Inc. (St. Louis, MO, USA). Aluminium chloride and L-ascorbic acid were purchased from Ajax Finechem (Taren Point, Australia). Methanol, trifluoroacetic acid and acetonitrile were purchased from Fisher Scientific (Thermo Fisher Scientific, Waltham, MA, USA).

### 4.2. Plant Materials and Extraction

*Ipomoea pes-caprae* (IPC) leaves were obtained from Dongtan Beach, Sattahip, Chonburi, Thailand and classified by Dr. Yuttaya Yuyen (Research and Development Institute, Suan Dusit University). A voucher specimen was deposited at Queen Sirikit Botanic Garden Herbarium under number M. Norsaengsri 5814. The leaves were cleaned with water, air-dried, and then frozen overnight at −80 °C in a Revco™ UxF −86 °C Upright Ultra-Low Temperature Freezer (Thermo Scientific™, Ireland). The frozen samples were freeze-dried using a FreeZone 6 Liter −84 °C Console Freeze Dryer with Stoppering Tray Dryer (Labconco, Kansas City, MO, USA). The freeze-dried powder of IPC leaves was extracted in ethanol, as previously described [44] with slight modification. Briefly, 100 g of dried IPC was macerated overnight in 2 L of 70% and 95% ethanol at room temperature. After three rounds of maceration, the alcoholic extracts were pooled, filtered, then evaporated under reduced pressure below 45 °C. The yields of 70% ethanolic (IPC70) and 95% ethanolic (IPC95) extracts were 25.22 and 17.27%, respectively.

### 4.3. Determination of Total Phenolic Content

The total phenolic compounds in IPC extracts were determined using the Folin–Ciocalteu reagent, as previously described [44]. Briefly, 4.5 µL of 1 mg/mL extracts and 126 µL of deionized water were added to 96-well plates, then mixed with 90 µL of 2% Na_2_CO_3_ for 3 min, and 4.5 µL of 50% Folin–Ciocalteu reagent were added. After the samples were incubated for 30 min, the resulting blue molybdenum-tungsten complex was detected at 750 nm using a microplate reader (Biochrom EZ Read 2000, Cambridge, UK). The total phenolic content in IPC extracts was quantified using a gallic acid standard and interpreted as milligrams of gallic acid equivalent to one gram of extract (mg/GAE/g extract).

### 4.4. Determination of Flavonoid Content

The flavonoid compounds of IPC extracts were detected using the aluminium chloride assay, as previously described [44]. Briefly, 100 µL of 1 mg/mL extracts were mixed with 100 µL of 2% AlCl_3_ in 96-well plates. The samples were then incubated for 30 min, and the absorbance at 415 nm was determined with a microplate reader (Biochrom EZ Read 2000, Cambridge, UK). The total flavonoid content of samples was calculated by comparison to the quercetin standard and interpreted as quercetin equivalent to one gram of extract (mg QE/g extract).

### 4.5. Detection of Tannin Content

Tannin content was determined using a modified method from that previously described [45,46]. In 96-well plates, 7.5 µL of 1 mg/mL extracts were diluted with 205 µL of deionized water and mixed with 25 µL of 35 mg/mL sodium bicarbonate and then 12.5 µL of Folin-Ciocalteu reagent was added. After the samples were incubated for 30 min, the absorbance was determined at 745 nm with a microplate reader (Biochrom EZ Read 2000, Cambridge, UK). The tannin content of the extracts was calculated against a tannic acid standard and interpreted as tannic acid equivalent to one gram of extract (mg TAE/g extract).

### 4.6. Phytochemical-Screening Assay by HPLC

The chemical composition of IPC extracts was analyzed utilizing high performance liquid chromatography (HPLC) using a modified method from previous reports [19,32,47]. HPLC was performed using Waters Alliance e2695 Separations Module, 2498 UV/Vis Detector with a C18 column (5 µm 4.6 × 250 mm, Waters Alliance), and data were analyzed with MassLynx software (Waters Alliance, USA). Crude extracts of IPC and standard solutions (chlorogenic acid, ferulic acid, 3,5-di-caffeoylquinic acid, and quercetin) were dissolved in methanol and acetonitrile (2:8, *v*/*v*) at a concentration of 1 mg/mL, filtered (0.2 mm, Millipore), and 10 µL of samples were injected. Solvents for HPLC analysis were acetonitrile as solvent A and 0.1% (*v*/*v*) trifluoroacetic acid in deionized water as solvent B at a flow rate of 1 mL/min. These experiments were run at 30 °C with the following gradients: 0–5% A linear (0–5 min), 5–10% A linear (5–10 min), 10% A linear (10–15 min), 10–15% A linear (15–20 min), 15–25% A linear (20–30 min), 25% A linear (30–35 min), 25–50% A linear (35–40 min), 50–80% A linear (40–50 min), and 80–100% A linear (50–60 min). Chromatograms were recorded at 360 nm, and quantitative determination of the compounds was analyzed using peak area with an external standard.

### 4.7. Determination of Antioxidant Activity

#### 4.7.1. 2,2-Diphenyl-1-picrylhydrazyl (DPPH) Radical-Scavenging Assay

Antioxidant activity of IPC extracts was detected using the DPPH-scavenging assay, as previously reported [44]. In 96-well plates, 75 µL of 0.0312–1 mg/mL extracts were mixed with 150 µL of 0.2 mM DPPH and incubated for 30 min. The absorbance was then determined at 515 nm by a microplate reader (Biochrom EZ Read 2000, Cambridge, UK). L-ascorbic acid and ethanol were the positive and blank controls, respectively. The antioxidant activity was interpreted as an IC_50_ value, the concentration of extract capable of scavenging 50% of DPPH radicals.

#### 4.7.2. 2,2′-Azinobis-(3-ethylbenzo-thiazoline-6-sulfonic acid) (ABTS) Radical-Scavenging Assay

The antioxidant capacity of IPC extracts was measured using the ABTS-scavenging assay [48]. Briefly, the ABTS solution was prepared by reacting 14 mM ABTS and 4.9 mM potassium persulfate at a ratio of 1:1 (*v*/*v*) for 16 h in the darkroom, and the mixture was diluted with distilled water (1:9, *v*/*v*) for the absorbance to reach 0.7 ± 0.02 at 734 nm. In 96-well plates, 50 µL of extracts at various concentrations (0.062–2 mg/mL) were mixed with 50 µL of ABTS solution. After 7 min of incubation at room temperature, the absorbance was measured at 734 nm using a microplate reader (Biochrom EZ Read 2000, Cambridge, UK). L-ascorbic acid was used as a positive control. The antioxidant activity was interpreted as an IC_50_ value.

#### 4.7.3. Ferric-Reducing Antioxidant Power (FRAP)

The antioxidant activity of the extracts was also assessed using an FRAP assay [49]. Briefly, the FRAP reagent was freshly prepared by mixing 300 mM acetate buffer (pH 3.6), 20 mM ferric chloride hexahydrate, and 10 mM 2,4,6-Tris (2-pyridyl)-s-triazine (TPTZ) in 40 mM HCl at a ratio 10:1:1 (*v*/*v*/*v*). In 96-well plates, 7.5 µL of 0.1–1 mg/mL extracts were mixed with 142.5 µL of the FRAP reagent and incubated for 4 min at 37 °C. The ferrous-tripyridyltriazine complex was detected at 593 nm using a microplate reader (Biochrom EZ Read 2000, Cambridge, UK). The FRAP activity was determined against a standard curve of Trolox. The FRAP values of extracts were expressed as µM Trolox equivalent to one gram of extract (µM TE/g extract).

### 4.8. Collagenase Activity Assay

Inhibition of collagenase activity was measured using a collagenase activity assay kit (Colorimetric, Abcam, USA). In 96-well plates, 2 µL of extracts at various concentrations (0.78–100 mg/mL), 88 µL of collagenase assay buffer and 10 µL of collagenase, were mixed and incubated for 10 min at 37 °C. One hundred microliters of collagenase substrate (FALGPA) were added, and the absorbance was measured immediately at 345 nm on a microplate reader in kinetic mode for at least 10–40 min at 37 °C. Solutions containing only collagenase and FALGPA were used as the negative control. One millimolar of 1,10-Phenanthroline was a collagenase inhibitor used as a positive control. The inhibition of collagenase activity was calculated and expressed as a percentage of the control. The IC_50_ for collagenase inhibition was calculated from a linear regression graph and is the concentration of extract capable of reducing collagenase activity to 50%.

### 4.9. Cell Viability Assay

Cell viability of human fibroblasts (CCD-986sk cells, ATCC CRL1947) was determined with PrestoBlue^®^ Cell Viability Reagent to exhibit the non-cytotoxic concentrations of IPC70 and IPC95 extracts [50]. CCD-986sk cells were cultured in Dulbecco′s Modified Eagle′s Medium (DMEM, Gibco^®^/Thermo Scientific, Waltham, MA, USA) with 10% fetal bovine serum (FBS) and 1% penicillin and streptomycin in an incubator with 5% CO_2_ at 37 °C. Briefly, 5000 cells/well were plated in 96-well plates and incubated overnight at 37 °C. The culture medium was removed, and then, the cells were treated for 72 h with 100 µL of extracts, diluted at various concentrations (0.0078–1 mg/mL). The culture medium with extracts was removed, and the treated cells were added with 90 µL of medium and 10 µL of Presto blue. After an additional 3 h, the absorbance was monitored at 570 and 600 nm using a MultiSkan Sky microplate reader (Thermo Fisher Scientific, Bremen, Germany). The results were interpreted as the percentage of cell viability by comparing with untreated cells as a control.

### 4.10. Cell Proliferation Assay

The effect of IPC70 and IPC95 extracts on cell proliferation was monitored with PrestoBlue^®^ Cell Viability Reagent [6,50]. Human fibroblast (CCD-986sk) cells were seeded at 5000 cells/well in 100 µL of DMEM with 10% FBS and 1% penicillin and streptomycin and incubated at 37 °C for 24 h. The FBS concentration was then lowered to 2% (*v*/*v*), and the cells were treated for 72 h with extracts at various concentrations (0.031–0.125 mg/mL) or 10 ng/mL of recombinant human TGF-β (rhTGF-β, R&D system, Minneapolis, MN, USA) as a control. After the culture medium was removed, the treated cells were added with 90 µL of medium and 10 µL of Presto blue and further incubated for 3 h. The absorbance was measured at 570 and 600 nm using a MultiSkan Sky microplate reader (Thermo Fisher Scientific, Bremen, Germany). The results were interpreted as the percentage of cell proliferation by comparing with untreated cells as a control.

### 4.11. In Vitro Scratch Wound Assay

To examine the skin regenerative capacities of IPC70 and IPC95 extracts by in vitro scratch wound assay, modified as previously described [50], human fibroblast (CCD-986sk) cells were seeded 50,000 cell/well in 24-well plates and cultured in 1 mL of DMEM with 10% FBS and 1% penicillin and streptomycin at 37 °C for 48 h to form confluent monolayers. After cell-free zones (scratches) were created using a wound-maker, each well was washed with phosphate buffer saline (pH 7.4). The cells were treated with 0.031 mg/mL of extracts or 10 ng/mL of rhTGF-β in a medium with 2% FBS. Cell migration was monitored every 24 h by phase-contrast imaging using an inverted light microscope Eclipse TS100 (Nikon, Tokyo, Japan). The cell-free or wound area was measured using the Image J program. The results were interpreted as the percentage of wound area by comparing with each condition at 0 h.

### 4.12. Detection of Type I Collagen and Expression of Collagen Type I (COL1A1), Tumor Growth Factor-Beta 1 (TGFB1), and Beta-Fibroblast Growth Factor (FGF2) Genes

Type I collagen was determined using Human Collagen, Type I, Alpha 1 (COL1A1) ELISA Kit (CUSABIO, www.cusabio.com, accessed on 15 October 2021). The expressions of COL1A1, TGFB1, and FGF2 genes was detected using real-time PCR [21,51]. Briefly, CCD-986sk cells, 50,000 cells/well in 24-well plates, were cultured in 1 mL of DMEM with 10% FBS and 1% penicillin and streptomycin at 37 °C for 24 h. After the culture medium was removed, the cells were treated with 0.031–0.062 mg/mL of extracts or 10 ng/mL of rhTGF-β in medium with 2% FBS at 37 °C for 72 h. The culture supernatants were collected for measuring type I collagen by ELISA. The treated cells were harvested for monitoring gene expression by real-time PCR.

The culture supernatants were monitored using type I collagen ELISA Kit, according to the manufacturer’s protocol (COL1A1 ELISA Kit, CUSABIO, www.cusabio.com, accessed on 15 October 2021) [20]. This assay employs the quantitative sandwich enzyme immunoassay. The antibody was specific for type I collagen that had been pre-coated onto a 96-well plate. Samples were bound by the immobilized antibody and captured with a biotin-conjugated antibody specific for type I collagen. The color development from the reaction of avidin-conjugated horseradish peroxidase and substrate was used to detect the amount of collagen bound in the initial step. The concentration of collagen was calculated from a linear regression graph of collagen standard.

RNA was extracted from different conditions of dermal fibroblast cells using the RNeasy kit (QIAGEN Sciences Inc., Germantown, MD, USA). The RNA concentration was measured at 230/260 nm using the MultiSkan Sky microplate reader (Thermo Fisher Scientific, Bremen, Germany). The cDNA synthesis was done using the Improm II kit (Promega, Madison, WI, USA). The gene expression was studied by Realtime PCR using TaqMan gene expression assay (Thermo Scientific, USA), including COL1A1 (Hs00164004_m1), TGFB1 (Hs00998133_m1), FGF2 (Hs00266645_m1), and GAPDH (Hs02786624_m1). Quantinova RT-PCR Master Mix Reagent (Qiagen, Hilden, Germany) was used. The PCR reactions were performed on the Azure Cielo Real-Time PCR system (Azure Biosystems, Dublin, CA, USA) following the manufacturer’s instructions. The resulting data were analyzed with Azure Cielo software.

### 4.13. Statistical Analysis

All grouped data were analyzed with SPSS version 17. Data were presented as mean ± standard deviation (SD) of three independent experiments. Statistical analyses were performed by *t*-test between two groups or one-way analysis of variance (one-way ANOVA) using Fisher’s Least Significant Difference (LSD) among more than two groups. *p* < 0.05 was considered to be statistically significant.

## 5. Conclusions

This study shows in vitro bioactivity of ethanolic extracts from *I. pes-caprae* leaves. The antioxidant and collagenase inhibitory properties of *I. pes-caprae* extracts were associated with 3,5-di-caffeoylquinic acid, chlorogenic acid, and ferulic acid. Ethanolic extracts of *I. pes-caprae* at noncytotoxic concentrations were found to promote cell proliferation, collagen production, and wound healing. Based on these findings, it may be possible to develop *I. pes-caprae* extracts as an effective anti-aging agent for use in cosmeceutical products to prevent skin aging.

## Figures and Tables

**Table 1:** Total phenolic, flavonoid, and tannin contents of IPC extracts.

Extracts	Total Phenolic Contents mg GAE/g Extract	Total Flavonoid Contents mg QE/g Extract	Total Tannin Contents mg TAE/g Extract
IPC70	75.315 ± 9.074	17.641 ± 0.966	18.39 ± 0.77 ^a^
IPC95	67.095 ± 6.202	26.815 ±1.133 ^a^	6.57 ± 0.68

Data are mean ± SD from three independent experiments. (^a^) indicates significant difference at *p* < 0.05.

**Table 2:** Antioxidant and collagenase inhibitory activities of IPC extracts.

Extracts	Antioxidant Activity			Collagenase Inhibitory Activity IC_50_ (mg/mL)/ (% of Inhibition)
	DPPH IC_50_ (mg/mL)	ABTS IC_50_ (mg/mL)	FRAP (µM TE/g Extract)	
IPC70	0.342 ± 0.021	0.259 ± 0.070 ^a^	170,427.67 ± 1325.24 ^a^	5.541 ± 0.044 ^a^
IPC95	0.403 ± 0.036	0.601 ± 0.057	53,530.40 ± 4587.89	12.179 ± 0.413
L-ascorbic acid	0.037 ± 0.002	0.034 ± 0.002	-	-
1,10-Phenanthroline (10 mM)	-	-	-	(100%)

Data are mean ± SD from three independent experiments. (^a^) indicates significant difference at *p* < 0.05 in comparing IPC70 and IPC95; IC_50_ is the concentration of samples that inhibited 50% of antioxidant activities.

## Data Availability

Not applicable.
